# Automated reporting of primaquine dose efficacy, tolerability and safety for *Plasmodium vivax* malaria using a systematic review and individual patient data meta-analysis

**DOI:** 10.1186/s12936-025-05642-w

**Published:** 2025-11-14

**Authors:** Peta Edler, Megha Rajasekhar, David J. Price, Ishag Adam, Ghulam Rahim Awab, Bridget E. Barber, Larissa W. Brasil, Nathália N. Chamma-Siqueira, Cindy S. Chu, Liwang Cui, André Daher, Margarete do Socorro M. Gomes, Lilia Gonzalez-Ceron, Matthew J. Grigg, Harin Karunajeewa, Marcus V. G. Lacerda, Simone Ladeia-Andrade, Toby Leslie, Benedikt Ley, Kartini Lidia, Alejandro Llanos-Cuentas, Rhea J. Longley, Laurens Manning, Daniel Abebe Mekonnen, Wuelton Marcelo Monteiro, Brioni R. Moore, Francois Nosten, Ayodhia Pitaloka Pasaribu, Dhelio B. Pereira, Jeanne Rini Poespoprodjo, Sasithon Pukrittayakamee, Komal Raj Rijal, Kavitha Saravu, André M. Siqueira, Inge Sutanto, Walter R. J. Taylor, Pham Vinh Thanh, Kamala Thriemer, José Luiz F. Vieira, Nicholas J. White, Asnakew K. Yeshiwondim, Lina M. Zuluaga-Idarraga, Philippe J. Guerin, Julie A. Simpson, Ric N. Price, Robert J. Commons

**Affiliations:** 1 Department of Infectious Diseases, The University of Melbourne at the Peter Doherty Institute for Infection and Immunity, Melbourne, VIC Australia; 2 Centre for Epidemiology and Biostatistics, Melbourne School of Population and Global Health, The University of Melbourne, Melbourne, VIC Australia; 3 Department of Obstetrics and Gynecology, College of Medicine, Qassim University, Buraidah, Saudi Arabia; 4 Mahidol Oxford Tropical Medicine Research Unit (MORU), Faculty of Tropical Medicine, Mahidol University, Bangkok, Thailand; 5 Department of Public and Global Health, Afghan International Islamic University, Kabul, Afghanistan; 6 QIMR Berghofer Medical Research Institute, Brisbane, QLD Australia; 7 Global and Tropical Health Division, Menzies School of Health Research and Charles Darwin University, Darwin, NT Australia; 8 Infectious Diseases Society Sabah-Menzies School of Health Research Clinical Research Unit, Kota Kinabalu, Sabah, Malaysia; 9 Diretoria de Ensino e Pesquisa, Fundação de Medicina Tropical Dr. Heitor Vieira Dourado, Manaus, Amazonas Brazil; 10 Programa de Pós‑Graduação em Medicina Tropical, Universidade do Estado do Amazonas, Manaus, Amazonas Brazil; 11 Laboratório de Malária, Instituto Evandro Chagas, Ministério da Saúde do Brasil, Ananindeua, Pará State Brazil; 12 Shoklo Malaria Research Unit, Mahidol Oxford Tropical Medicine Research Unit, Faculty of Tropical Medicine, Mahidol University, Mae Sot, Thailand; 13 Centre for Tropical Medicine and Global Health, Nuffield Department of Clinical Medicine, University of Oxford, Oxford, UK; 14 Department of Internal Medicine, Morsani College of Medicine, University of South Florida, Tampa, FL USA; 15 Fiocruz Clinical Research Platform, Oswaldo Cruz Foundation (FIOCRUZ), Rio de Janeiro, Brazil; 16 Vice‑Presidency of Research and Biological Collections, Oswaldo Cruz Foundation (FIOCRUZ), Rio de Janeiro, Brazil; 17 Superintendência de Vigilância em Saúde do Estado do Amapá - SVS/AP, Macapá, Amapá Brazil; 18 Federal University of aMAPA, Universidade Federal do Amapá - UNIFAP), Macapá, Amapá Brazil; 19 Regional Centre for Public Health Research, National Institute for Public Health, Tapachula, Chiapas, Mexico; 20 Department of Medicine-Western Health, Melbourne Medical School, The University of Melbourne, St. Albans, VIC Australia; 21 Fundação de Medicina Tropical Dr Heitor Vieira Dourado, Manaus, Brazil; 22 Instituto Leônidas E Maria Deane, Fiocruz, Manaus, Brazil; 23 University of Texas Medical Branch, Galveston, USA; 24 Laboratory of Parasitic Diseases, Oswaldo Cruz Institute, Fiocruz, Rio de Janeiro, Brazil; 25 Institute of Hygiene and Tropical Medicine, Global Health and Tropical Medicine, NOVA University of Lisbon, Lisbon, Portugal; 26 Department of Infectious and Tropical Diseases, London School of Hygiene and Tropical Medicine, London, UK; 27 HealthNet-TPO, Kabul, Afghanistan; 28 The Department of Pharmacology and Therapy, Faculty of Medicine and Veterinary Medicine, Universitas Nusa Cendana, Kupang, Indonesia; 29 Unit of Leishmaniasis and Malaria, Instituto de Medicina Tropical “Alexander Von Humboldt”, Universidad Peruana Cayetano Heredia, Lima, Peru; 30 Mahidol Vivax Research Unit, Faculty of Tropical Medicine, Mahidol University, Bangkok, Thailand; 31 Infection and Global Health Division, Walter and Eliza Hall Institute of Medical Research, Melbourne, Australia; 32 Department of Medical Biology, University of Melbourne, Melbourne, Australia; 33 Medical School, University of Western Australia, Crawley, Australia; 34 Wesfarmers Centre of Vaccines and Infectious Diseases, Telethon Kids Institute, Perth, Australia; 35 Department of Infectious Diseases, Fiona Stanley Hospital, Perth, Australia; 36 Malaria and Neglected Tropical Diseases Directorate, Armauer Hansen Research Institute, Addis Ababa, Ethiopia; 37 Haramaya University, Dire Dawa, Ethiopia; 38 Universidade do Estado do Amazonas, Manaus, Brazil; 39 Curtin Medical School, Faculty of Health Science, Curtin University, Perth, Australia; 40 Curtin Medical Research Institute, Curtin University, Perth, Australia; 41 Department of Pediatrics, Medical Faculty, Universitas Sumatera Utara, Medan, North Sumatera Indonesia; 42 Centro de Pesquisa Em Medicina Tropical de Rondonia (CEPEM), Porto Velho, Brazil; 43 Fundação Universidade Federal de Rondonia (UNIR), Porto Velho, Brazil; 44 Mimika District Hospital, Timika, Indonesia; 45 Timika Malaria Research Programme, Papuan Health and Community Development Foundation, Timika, Indonesia; 46 Paediatric Research Office, Faculty of Medicine, Public Health and Nursing, Universitas Gadjah Mada, Yogyakarta, Indonesia; 47 Central Department of Microbiology, Tribhuvan University, Kirtipur, Nepal; 48 Department of Clinical Tropical Medicine, Faculty of Tropical Medicine, Mahidol University, Bangkok, Thailand; 49 Department of Infectious Diseases, Kasturba Medical College Manipal, Manipal Academy of Higher Education, Manipal, Madhava Nagar, Manipal, Karnataka India; 50 Instituto Nacional de Infectologia Evandro Chagas, Fundação Oswaldo Cruz (Fiocruz), Rio de Janeiro, Brazil; 51 Department of Parasitology, Faculty of Medicine, University of Indonesia, Jakarta, Indonesia; 52 Parasitology and Entomology, National Institute of Malariology, Hanoi, Vietnam; 53 Federal University of Pará (Universidade Federal Do Pará - UFPA), Belém, Pará, Brazil; 54 World Health Organization Country Office for Addis Ababa, Addis Ababa, Ethiopia; 55 Grupo Malaria, Facultad de Medicina, Universidad de Antioquia, Medellín, Colombia; 56 Facultad Nacional de Salud Publica, Universidad de Antioquia, Medellín, Colombia; 57 WorldWide Antimalarial Resistance Network (WWARN), Oxford, UK; 58 Infectious Diseases Data Observatory (IDDO), Oxford, UK; 59 WorldWide Antimalarial Resistance Network (WWARN), Asia-Pacific Regional Centre, Melbourne, Australia; 60 General and Subspecialty Medicine, Grampians Health—Ballarat, Ballarat, Australia; 61 ICAP, Columbia University Mailman School of Public Health, Addis Ababa, Ethiopia; 62 International Centre for Diarrhoeal Disease Research, Dhaka, Bangladesh; 63 Malaria and Other Parasitic Disease Research Team, Ethiopian Public Health Institute, Addis Ababa, Ethiopia; 64 Institute for Global Health and Infectious Diseases, University of North Carolina at Chapel Hill, Chapel Hill, NC USA; 65 Oxford University Clinical Research Unit Indonesia, Jakarta, Indonesia; 66 Royal Brisbane and Women’s Hospital, Brisbane, Australia; 67 Oxford University Clinical Research Unit, Hospital for Tropical Diseases, Ho Chi Minh City, Vietnam; 68 College of Medicine and Health Sciences, Arbaminch University, Arbaminch, Ethiopia; 69 Disease Control and Elimination Theme, Medical Research Council Unit The Gambia at LSHTM, Serrekunda, The Gambia; 70 Department of Parasitology, Institute of Biomedical Sciences, University of São Paulo, São Paulo, Brazil; 71 Department of Pathology and Laboratory Medicine, Aga Khan University, Karachi, Pakistan; 72 Formerly Senior Director, Global Health, GlaxoSmithKline, Brentford UK; 73 Health Protection and Research Organisation, Kabul, Afghanistan; 74 U.S. President’s Malaria Initiative, Malaria Branch, U.S. Centers for Disease Control and Prevention, Atlanta, GA USA; 75 Department of Infectious Diseases, Northwick Park Hospital, Harrow, UK; 76 Department of Medicine, Kasturba Medical College, Manipal University, Madhav Nagar, Manipal, Karnataka India; 77 ICMR-Rajendra Memorial Research Institute of Medical Sciences, Patna, Bihar India; 78 College of Health Sciences, Addis Ababa University, Addis Ababa, Ethiopia; 79 University of Gondar, Gondar, Ethiopia; 80 Department of Human Biology, University of Melbourne, Melbourne, Australia; 81 Secretaria Estadual de Saúde do Acre- Enfermaria e Ambulatório de Doenças Infecciosas, Acre, Brazil; 82 Afya Faculdade de Ciências Médica Cruzeiro do Sul, Cruzeiro do Sul, Brazil; 83 Faculty of Medicine, Universitas Indonesia, Jakarta, Indonesia; 84 Division of Tropical Medicine and Infectious Disease. Department of Internal Medicine, Cipto Mangunkusumo Hospital, Jakarta, Indonesia; 85 Universidade Federal Fluminense (UFF), Rio de Janeiro, Brazil; 86 Centro de Pesquisa, Diagnóstico e Treinamento Em Malária (CPD-Mal) da Fiocruz e da Secretaria de Vigilância em Saúde e Ambiente, Ministério da Saúde, Brasília, Brazil; 87 Coordenação de Pesquisa, Instituto Nacional de Câncer, Rio de Janeiro, Brazil; 88 Centro Universitário do Pará - CESUPA, Belém, Pará State Brazil; 89 Programa de Pós-Graduação em Biologia de Agentes Infecciosos e Parasitários, Belém, Pará State Brazil; 90 Infectious Diseases Unit, Clinical Research Centre, Queen Elizabeth Hospital, Kota Kinabalu, Sabah, Malaysia; 91 Subang Jaya Medical Centre, Subang Jaya, Malaysia; 92 Department of Infectious Diseases, Royal Perth Hospital, Perth, Australia; 93 Ethiopian Public Health Institute, Addis Ababa, Ethiopia; 94 Arbaminch University, Arbaminch, Ethiopia

**Keywords:** *Plasmodium vivax*, Malaria, Primaquine, Recurrence, Relapse, Dose, Efficacy, Tolerability, Haemolysis, Automated report

## Abstract

**Background:**

The antirelapse efficacy of primaquine is related to the total dose administered, whereas the risks of haemolysis and gastrointestinal intolerance are associated with the daily dose administered. National Malaria Control Programmes require local information on efficacy, tolerability and safety to optimize antimalarial treatment policies for *Plasmodium vivax* malaria control and elimination efforts.

**Methods:**

A living systematic review identified efficacy studies of uncomplicated *P. vivax* malaria including patients treated with daily primaquine regimens, published since January 1, 2000. Available data were pooled and an R Shiny app was developed to integrate statistical analyses performed using R and Stata that assessed the impact of primaquine mg/kg dose on efficacy, hematological safety and gastrointestinal tolerability.

**Results:**

As of January 16, 2025, a total of 9,270 individual patient data records from 41 studies have been collated into the standardized repository. Open-access automated reports were generated for user-selected countries or regions to investigate location specific effects of primaquine dose on efficacy, safety and tolerability. The reports include visual and tabular displays of the outcomes.

**Conclusions:**

These automated reports leverage a large database to provide accessible data for national and regional policy makers and researchers to assess the clinical consequences of different primaquine regimens in different endemic settings. The reports will inform local and regional policy decisions and research priorities in vivax-endemic areas.

## Background

Efforts to reduce malaria transmission in countries co-endemic for *Plasmodium falciparum* and *Plasmodium vivax* have been much more successful for *P. falciparum* [1]. *P. vivax* forms dormant liver stages (hypnozoites) that cause relapsing infections, contributing to 66–90% of all recurrent episodes of vivax malaria, sustaining ongoing transmission and hampering *P. vivax* elimination [2, 3]. Killing hypnozoites requires treatment with an 8-aminoquinoline drug, of which primaquine is the most widely available. The antirelapse efficacy of primaquine is related to the total dose administered, however, in practice a major barrier to its use are concerns of haemolysis in individuals with glucose-6-phosphate dehydrogenase (G6PD) deficiency, which is related to the daily dose administered [4, 5]. Single-dose tafenoquine has been recently recommended by the World Health Organization (WHO) for use in South America [6], however, to date, concerns about its optimal dose [7] and its use in combination with artemisinin-based combination therapy [8] have slowed widespread implementation.

Primaquine doses are generally given as 3.5 mg/kg (low) or 7 mg/kg (high) total dose regimens over 7 or 14 days, equating to 0.25 mg/kg (low), 0.5 mg/kg (intermediate) or 1 mg/kg (high) per day. Previous guidelines recommend low total dose primaquine regimens (3.5 mg/kg over 7 or 14 days), which were considered to provide sufficient efficacy in most vivax-endemic settings since higher daily doses are associated with an increased risk of haemolysis and gastrointestinal intolerability [9]. However, comparative data informing these guidelines have been sparse. Recent individual patient data meta-analyses undertaken by the WorldWide Antimalarial Resistance Network (WWARN) have used data from 23 studies conducted in 16 vivax-endemic countries across the Americas, Africa, and the Asia–Pacific [10, 11], and demonstrated that increasing the total dose of primaquine from 3.5 to 7 mg/kg could potentially reduce the risk of vivax recurrence within 180 days by approximately 50% [10]. High total dose primaquine requires higher daily mg/kg dosing, but patients treated with primaquine doses of up to 0.5 mg/kg/day had a minimal increase in gastrointestinal intolerance with no increased risk of severe hemolysis in individuals with ≥ 30% G6PD activity [10, 11].

National Malaria Control Programmes (NMCPs) have highlighted the importance of regional and subregional data on primaquine efficacy and safety, since these may differ from the global pooled results. NMCPs have requested these data to inform decisions regarding national antimalarial policies [12]. To assist regional and national policy makers to access these data, we developed open access, automated local and regional reports using a single standardized data repository. This manuscript aims to describe the automated process of generating these reports and highlight their role for policy makers.

## Methods

Studies were identified as part of an existing living systematic review of efficacy studies of uncomplicated *P. vivax* published since January 1, 2000 in any language. All studies included treatment with a daily primaquine regimen which was commenced within 7 days of schizontocidal treatment and had a minimum follow up duration of 28 days [10, 11, 13]. Investigators of eligible studies were approached to share individual patient data. Pseudo-anonymized data were shared to the WWARN repository, standardized and pooled into a single database according to an a priori statistical analysis plan. Patients were excluded from the analysis if they presented with severe malaria, received adjunctive antimalarials within 14 days of commencing treatment, received an alternative hypnozoiticidal agent to primaquine, received primaquine 7 or more days after commencing their schizontocidal treatment, had a protocol violation in the original study, or were missing age, sex or primaquine dose.

Efficacy analyses assessed the total dose of primaquine, and tolerability and safety analyses assessed the daily dose of primaquine. The total dose of primaquine regimens was categorized into two bands: low (2– < 5 mg/kg total dose) and high dose (≥ 5 mg/kg total dose), reflecting standard 3.5 mg/kg and 7 mg/kg total dose regimens. Patients with doses < 2 mg/kg were excluded from the efficacy analyses. Daily primaquine dose was categorized into three bands: low (< 0.375 mg/kg/day), intermediate (0.375– < 0.75 mg/kg/day) and high dose (≥ 0.75 mg/kg/day), reflecting standard 0.25 mg/kg, 0.5 mg/kg and 1 mg/kg daily dose regimens.

**Fig. 1:**
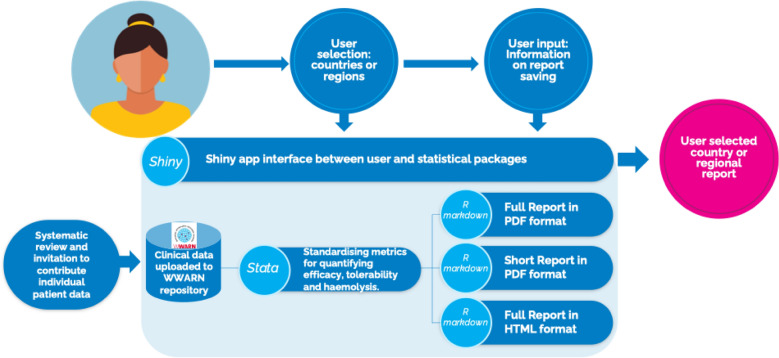
Generation of region and country automated reports on primaquine efficacy and safety

The statistical software packages R and Stata and the notebook interface R Markdown were used to generate reports on the effects of primaquine mg/kg dosing on efficacy, haematological safety and gastrointestinal tolerability. An R Shiny app was developed to integrate the statistical packages and provide a user interface to generate open-access, user-selected, country or regional automated reports in a variety of formats (Fig. 1).

The statistical analyses for the reports were based on a priori statistical analysis plans developed from the previous meta-analyses [10, 11], in which data could be filtered by country or region, based on the user selection in the R Shiny app. For each model, simple checks were undertaken, to ensure availability of reference data, that confidence intervals were logical, and that models converged (as detailed below).

Kaplan–Meier survival analysis was used to calculate the risk of recurrence between day 7 and day 365 after starting primaquine treatment. Patients were left-censored on day 7 and right-censored on the day last reviewed, the last day before a > 60-day gap in blood smears, or the day of non-*P. vivax* parasitemia. The rate of vivax recurrence between day 7 and 180 in patients following low and high total dose primaquine compared with treatment without primaquine were estimated using Cox regression models adjusting for sex, age and baseline parasitemia, with shared frailty for study site. Each site within a study was considered to be a separate study site. Additional Cox models investigated the rate of vivax recurrence following 14-day primaquine treatment compared with 7-day treatment in patients with low or high total dose primaquine.

Patients were excluded from the tolerability analyses if they did not have symptom questionnaire data on tolerability, they received primaquine more than 7 days after commencing their schizontocidal treatment or they did not have data on daily primaquine dose. Gastrointestinal intolerance on days 5–7 was assessed according to the daily primaquine dose category using a logistic mixed-effects model, adjusting for age, sex and baseline parasite density, with a random effect for study site. Days 5–7 were chosen to avoid confounding from symptoms relating to schizontocidal treatment or acute malaria.

Patients were excluded from the hematological safety analyses if they did not have data on hemoglobin at day 0 and at least one follow up day, they received primaquine more than 7 days after commencing their schizontocidal treatment, they did not have data on daily primaquine dose and had unknown or < 30% G6PD activity. Linear mixed-effects regression was used to assess the absolute change in hemoglobin from day 0 to day 2–3 for each daily primaquine dose category, adjusting for hemoglobin on day 0, age, sex and baseline parasite density, with a random effect for study site. Day 2–3 was chosen as this is the time of expected hemoglobin nadir and when the majority of patients will be tested.

## Results

**Fig. 2:**
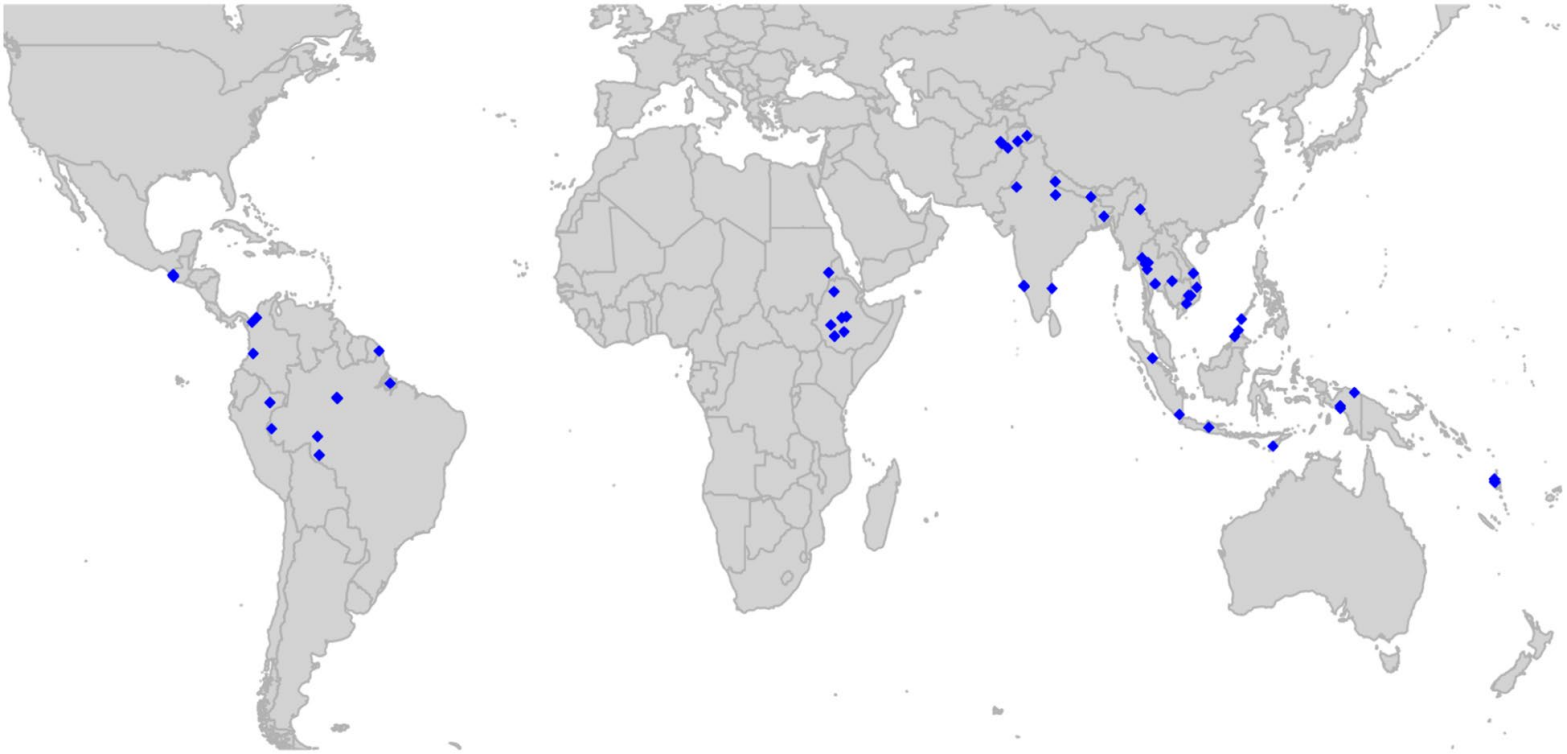
Map of study sites from which data were available for automated reports as of January 16, 2025

Of 108 eligible studies, 40 have been shared to the standardized repository, in addition to two unpublished studies. As of January 16, 2025, a total of 9,270 patients from 41 studies [14–53] have undergone curation and are available for inclusion in the analyses (Additional file 1: Fig. S1, Tables S1-2). There are 6346 (68.5%) patients from the Asia–Pacific, 1236 (13.3%) from Africa and 1,688 (18.2%) from the Americas (Fig. 2). In total, 1582 (17.1%) patients were treated without primaquine, 66 (0.7%) with very low total dose primaquine (< 2 mg/kg; these participants were excluded from efficacy analyses), 4282 (46.2%) with low total dose primaquine (2– < 5 mg/kg) and 3340 (36.0%) with high total dose primaquine (≥ 5 m g/kg). Data are currently available for 7508 patients from 27 studies for efficacy analyses, 5772 patients from 17 studies for tolerability analyses and 5,710 patients from 19 studies for hematological safety analyses.

The R Shiny app can generate R Markdown reports for three global regions (Africa, the Americas and Asia–Pacific) or any combinations of countries. The current country reports are grouped into 8 subregional groupings based on geographic location and study size. Reports can be generated as either a detailed or summary version, both of which can be provided in an html and pdf format to aid with accessibility (Fig. 1). The reports were updated following feedback from NMCPs through the Asia–Pacific Malaria Elimination Network. To ensure ease of access for users, privacy of data and accuracy of results, reports generated through the R Shiny app have been reviewed by authors and made openly accessible through the WorldWide Antimalarial Resistance Network. Reports are available for review and download at https://www.iddo.org/wwarn/vivax-reports.

The living systematic review will be updated every 6 months for the next five years, with studies eligible for the primaquine database to be identified. If available, data will be pooled into the standardized database and updated reports will be generated. This ongoing process has subsequently identified investigators from the 13 eligible studies published since the original database collation on August 23, 2021, who have been approached to share data; 3 of whom have provided data, and 2 others who have agreed to share data.

## Discussion

Automated reports for 17 countries and three global regions have been generated to define the effect of primaquine dose in patients with uncomplicated *P. vivax* malaria on efficacy, tolerability and safety; these are openly available using an existing global database of primaquine efficacy clinical trials. The WWARN global individual patient database will be updated twice yearly to provide countries and regions with up-to-date reports on efficacy and safety of primaquine as countries progress towards malaria elimination.

*Plasmodium vivax* strains differ between geographic regions with substantial heterogeneity in transmission, relapse phenotype, hypnozoite load and primaquine efficacy. For instance, regions such as Papua, Indonesia and Papua New Guinea have the rapidly relapsing variant (3–4 weeks between relapses), whereas relapses in South Asia generally occur 8–9 months after the initial infection [54]. In some areas, high antirelapse efficacy is achieved with relatively low total doses (3.5 mg/kg) of primaquine [21] whereas in other areas a higher total dose is required [10]. The availability of the automated reports using local data from countries and regions will guide policymakers to optimize primaquine dosing for vivax radical cure in their local context. As single-dose tafenoquine is implemented into national policies, there will be an opportunity to integrate efficacy, tolerability and safety data from tafenoquine efficacy studies into the automated reports and allow comparison with primaquine regimens.

Reports are limited by inclusion of only a subset of published data. Of the potentially eligible studies, 66 (61%) were unavailable for analysis, with most (48/66; 73%) published more than 10 years ago. Timely access to clinical data is critical for ensuring that reports are contemporary, and yet time lags between completion of trials and access and curation of data are an issue to overcome; only one of the 12 studies published since 2023 is available for the current reports. All investigators of these trials have been approached and two have agreed to share their data. One investigator has recently shared their data, which, along with data from an additional older study, will be added to the database in the coming months.

The methodology of trials assessing the antirelapse efficacy of 8-aminoquinolines is heterogeneous. Differences in the duration of follow up and inability to distinguish accurately between relapses, new infections and recrudescences, have hampered comparisons between trials, although new methods to distinguish recurrences have been developed [55, 56]. Similarly, there are a lack of standardized metrics to assess safety following 8-aminoquinoline treatment. The definition of hemolytic adverse events attributable to 8-aminoquinolines varies substantially between studies, and this can result in over- or under-estimation of the safety of 8-aminoquinolines. Defining severe drug-induced hemolysis attributable to 8-aminoquinolines is further complicated by associated parasite-induced hemolysis and the fall in hemoglobin being strongly correlated to the baseline hemoglobin concentration [57]. These automated reports provide standardized definitions for relapse efficacy and safety, ensuring results can be compared between trials and between geographic locations.

Automation of reports provide consistent safety and efficacy metrics across different study designs, and this will reduce the time to review and compare outputs from different studies. However, these reports are not formal analyses and therefore may lack detailed assessment of limitations that could potentially lead to misinterpretation. Users should be aware that there can be substantial variations in reporting of gastrointestinal symptoms [58] and that comparisons of non-randomized study arms may lead to unexpected findings. Administration of primaquine with food has been demonstrated to greatly reduce the risk of gastrointestinal symptoms [59]. The risk of bias assessments that are now routinely included in formal individual patient data meta-analyses have not been incorporated into the automated reports. Furthermore, users need to be aware of the potential for bias with efficacy studies with smaller sample sizes and consider these automated reports in the context of more formal published individual patient data meta-analyses [10, 11]. Although reports have been designed to automate analyses of data from published efficacy studies, they could also incorporate and analyse shared data from unpublished therapeutic efficacy studies conducted either by NMCPs or researchers, facilitating formal analysis and reporting. Queries about reports are available through a contact email at https://www.iddo.org/wwarn/vivax-reports.

Recent global evidence on primaquine mg/kg dosing suggests that higher total primaquine doses will lead to substantial reductions in *P. vivax* relapses across most endemic regions [6, 10]. The availability of national and regional reports describing the effect of primaquine mg/kg dose for uncomplicated *P. vivax* on efficacy and safety provides NMCPs with the opportunity to consider these global implications together with data from their local regions.

## Data Availability

The data are available for access via the WorldWide Antimalarial Resistance Network (WWARN.org). Requests for access will be reviewed by a Data Access Committee to ensure that use of data protects the interests of the participants and researchers according to the terms of ethics approval and principles of equitable data sharing. Requests can be submitted by email to malariaDAC@iddo.org via the Data Access Form available at WWARN.org/accessing-data. The WWARN is registered with the Registry of Research Data Repositories (re3data.org).
